# Effects of Dietary Fiber and Copper on the Performance and Gut Microbiota of Finishing Pigs

**DOI:** 10.3390/ani14223168

**Published:** 2024-11-06

**Authors:** Bo Liu, Jun Yan, Houxu Hao, Feng Yong, Lianyu Yang, Wenyan Yang, Dongsheng Che

**Affiliations:** 1 College of Animal Science and Technology, Jilin Agricultural University, Changchun 130118, China; 13166884099@163.com (B.L.); 13894160329@163.com (H.H.); yongfeng0485@163.com (F.Y.); yangly2004@126.com (L.Y.); 2 Key Laboratory of Animal Production, Product Quality and Security, Ministry of Education, Changchun 130118, China; 3 Jilin Provincial Key Laboratory of Animal Nutrition and Feed Science, Changchun 130118, China

**Keywords:** dietary fiber, trace element copper, finishing pigs, production performance, colon microflora

## Abstract

This study aimed to investigate the effects of dietary fiber (DF) levels and copper concentrations on the production performance and cecal microbial diversity of finishing pigs. A 2 × 2 factorial experimental design was used, with different levels of dietary fiber (low [23% DF]: L and high [30% DF]: H) and copper concentrations (normal [25 mg/kg]: N and supplemented [45 mg/kg]: S) resulting in four diets (LN, LS, HN, and HS). Forty-eight hybrid barrows (Duroc × Landrace × Yorkshire), with an initial body weight of 76 kg ± 1.5 kg, were randomly assigned to four groups: LN, LS, HN, and HS, with 12 replicates per group and one pig per replicate. There was a 7-day adaptation period followed by a 56-day feeding trial, after which all pigs were slaughtered for sampling. Results indicated that in finishing pigs, the low dietary fiber group exhibited a higher final weight, a higher average daily gain, and a lower feed-to-gain ratio compared to the high fiber group (*p* < 0.05). The LS group showed higher digestibility of dry matter, crude protein, crude fiber, ash, neutral detergent fiber, and DF than the HN and HS groups (*p* < 0.05). Blood total protein levels were higher in the high fiber group, whereas blood Cu levels were higher in the supplemented copper group (*p* < 0.05). High dietary fiber increased the activities of colonic carboxymethylcellulase and β-glucanase (*p* < 0.05). Concentrations of acetic acid, propionic acid, and total volatile fatty acids were elevated in the high fiber group (*p* < 0.05). Microbial α-diversity indices (observed species, Chao 1, and Shannon indices) increased with fiber but decreased with copper supplementation (*p* < 0.05). The *Firmicutes*/*Bacteroidetes* ratio increased with fiber levels, with a higher relative abundance of Lactobacillus in the LS group. In conclusion, appropriate copper supplementation in diets can mitigate the negative effects of high fiber levels on finishing pig production performance by enhancing nutrient digestibility, fiber-degrading enzyme activity, regulating the microbial community, and its metabolic products.

## Simple Summary

To reduce environmental pollution, the addition of copper in diets has been restricted. Meanwhile, the relatively high fiber (use of food processing by-products) content in finishing pig diets reduces the biological value of dietary copper. Therefore, under different fiber levels, the appropriate dietary copper supplementation should be reconsidered, which is crucial for further improving the production performance of finishing pigs. This study systematically evaluated the effects of dietary fiber and copper on the production performance and gut microbiota of finishing pigs to elucidate the growth-promoting effects of copper under varying dietary fiber levels.

## 1. Introduction

Dietary fiber and copper have different effects on swine production performance and intestinal health. Appropriate copper can promote the growth performance and health status of pigs by serving as a substrate for the synthesis of hemoglobin and oxidative enzymes necessary for normal metabolism [1]. Suitable levels of dietary fiber can act as a prebiotic without affecting animal growth, promoting the colonization of beneficial bacteria and the release of their metabolites, thereby improving immune responses and promoting intestinal health. However, excessive fiber can inhibit digestive enzyme activity, reducing nutrient utilization efficiency, which poses a challenge for the efficient utilization of fibrous feed ingredients in finishing pigs [2]. Although the Nutrient Requirements of Swine (NRC, 2012) provides basic recommendations for copper requirements at different physiological stages, previous studies have shown that increasing dietary copper levels can improve the growth performance of piglets and growing pigs, as well as the reproductive performance of sows [3]. This indicates that supplementary copper, beyond the basic requirement, has additional nutritional and functional effects. It is hypothesized that the growth-promoting effect of copper is partly due to its influence on the repair of intestinal tissue and its ability to improve the synthesis of digestive enzymes, thus improving nutrient digestion and absorption [4]. For instance, adding 250 mg/kg of copper to the diet of weaned piglets increased the activity of lipase and phospholipase A, thereby improving fat digestibility [5]. Gonzales-Eguia et al. found that increasing copper levels in the diet of growing pigs improved the digestibility of organic matter and fat [6]. Additionally, research by Lillie and Frobish demonstrated that increasing dietary copper levels to 60 mg/kg in sows improved the birth weight and weaning weight of piglets [7]. Another study reported that sows fed 9.5 mg/kg copper, compared to 2.0 mg/kg, had higher plasma ceruloplasmin concentrations and lower stillbirth rates. However, the effects of copper on finishing pigs have been less consistent [8]. Recent studies have indicated that adding 150 mg/kg of copper to the diets of late-stage finishing pigs increased the average daily feed intake (ADFI) and feed-to-gain ratio (F/G) [9]. This may be related to the fact that the diets of most piglets and growing pigs are based on corn–soybean meal, while finishing pig diets include more by-products of grain processing, altering the fiber level and structure. Research suggests that the adsorption effect of dietary fiber carries copper ions to the large intestine, and as dietary fiber is fermented and degraded by gut microbiota, some copper ions are re-released, promoting their dissociation and absorption [10]. In summary, this study hypothesizes the appropriate increase in dietary copper levels can alleviate the negative effects of high dietary fiber on swine production performance, thereby enhancing the biological value of copper. Therefore, this study aims to investigate the effects of dietary fiber and copper levels on the growth performance, nutrient utilization, carcass traits and meat quality, as well as gut microbiota of finishing pigs, and their interaction effects.

## 2. Materials and Methods

### 2.1. Experimental Design, Animals, and Diets

A 2 × 2 factorial treatment arrangement was explored for this study, with the main factors being 2 dietary fiber and dietary copper levels. Forty-eight finishing barrows (Duroc × Landrace × Yorkshire) with similar body weight (76.0 ± 1.5 kg) were randomly allotted into 4 dietary treatment groups. Four dietary treatment groups were namely, (1) low dietary fiber and normal dietary copper (LN, dietary fiber: 23%; dietary copper: 25 mg/kg); (2) low dietary fiber and supplemental dietary copper (LS, dietary fiber: 23%; dietary copper: 45 mg/kg); (3) high dietary fiber and normal dietary copper (HS, dietary fiber: 30%; dietary copper: 25 mg/kg); and (4) high dietary fiber and supplemental dietary copper (HN, dietary fiber: 30%; dietary copper: 45 mg/kg). The actual measured values of nutrients in all feedstuffs are shown in Table 1. The experimental diets and their nutritional compositions are presented in Table 2. All experimental diet formulations followed the recommendations of NRC (2012) for the nutritional requirements of finishing pigs and maintained relative consistency in digestible energy (DE) and crude protein (CP).

### 2.2. Feeding Management

During the study, all pigs had ad libitum access to water and feed, and they were individually housed in separate pens within the same pigsty. The experiment lasted for 63 days, including 7 days of pre-feeding and 56 days of experiment. The pigs were provided with unrestricted access to water and diets throughout the 56-day feeding period.

### 2.3. Slaughter Procedure and Sample Collection

One week before the experiment concluded, feces from all pigs were collected using a total collection method, with samples taken each morning and evening, and hydrochloric acid was used to fix nitrogen. These samples were then stored at −20 °C for later analysis of nutrient digestibility. At the end of the experiment, a 20 mL blood sample was taken from the anterior vena cava of the fasting finishing pigs. The serum was separated by centrifuging at 3000× *g* and 4 °C for 10 min. Post centrifugation, the plasma was portioned and stored at −80 °C for additional analyses.

The pigs were slaughtered at the Charoen Pokphand Food Co., Ltd. abattoir in Changchun, Jilin, located 32 km from the experimental site, requiring approximately 40 min of transportation. The slaughtering process adhered to commercial standards, involving electrocution for euthanasia, followed by exsanguination, dehairing, skinning, eviscerating, and splitting along the midline. From the left side, 100 g samples of LT were taken between the 9th and 10th ribs for chemical composition analysis and stored at −80 °C, while LT samples between the 11th and 14th ribs were used to assess meat quality. Additionally, colonic digesta was collected and divided into four 5 mL tubes, which were then frozen at −80 °C to evaluate cellulase activity and volatile fatty acid concentrations.

### 2.4. Chemical Analysis

Concentrations of dry matter (DM; method 930.15) and CP (method 954.01) in feedstuffs and fecal samples were measured according to the procedures of the Association of Official Analytical Chemists (2000). The concentrations of crude fiber (CF; method 978.10), ether extract (EE; method 920.39), ash (method 924.05), total calcium (method 984.01), and total phosphorus (method 965.17) in the feedstuffs samples were measured according to the procedures of the Association of Official Analytical Chemists (2006). Acid detergent fiber (ADF) and neutral detergent fiber (NDF) in the feedstuffs and fecal were determined using a fiber analyzer, according to a previous report [11].

### 2.5. Growth Performance and Nutrient Digestibility

The initial and final body weights (BWs) of all pigs, as well as feed intake per pen, were recorded to evaluate the average daily gain (ADG), average daily feed intake (ADFI), and feed-to-weight gain ratio (F:G). Calculation formula: ADG = (average weight at the end of the pen − average weight at the beginning of the pen)/(number of trial days × number of pens); ADFI = total feed intake per pen/(number of trial days × number of pens); and F:G = ADFI/ADG. Nutrient digestibility was determined according to previous reports. The calculation formula is apparent total tract digestibility (ATTD) = 100 × [nutrient intake (g/d) − nutrient excretion (g/d)]/nutrient intake (g/d).

### 2.6. Carcass Traits

After slaughter, several measurements were performed following the Technical Regulations for Performance Determination of Lean Breeding Pigs in China (GB8467-87). These included dressing percentage (DP), calculated as hot carcass weight (HCW) divided by live weight (LW) multiplied by 100%. Carcass oblique length (COL) was measured from the phalanx joint to the first rib intersection of the sternum (cm) [12]. Backfat thickness (BT) was determined by averaging the shoulder, penultimate rib, and lumbar spine thickness along the midline of the pig carcass (mm). The loin-eye area (LA) was calculated as the product of plane height, plane width, and 0.7 (cm^2^).

### 2.7. Meat Quality

The color attributes including lightness (L*), redness (a*), and yellowness (b*) were measured on the cross-section (3 locations) of the LT (the last rib) for each pig (24 h post-slaughter), using a MiniScan EZ (Model: 4500L, 45°/0° illumination system, Hunter Lab Corp., New York, NY, USA) calibrated against a standard white plate (measurement aperture: 31.8 mm, illuminant: D65, standard observer angle: 10°), with color development time after cutting being 10–15 min. After calibrating with pH 4.6 and 7.0 buffers, a pH meter (pH-STAR, SFK-Technology, Herlev, Denmark) was used to measure the pH value of LT muscle samples from the left side of the carcass at the last rib at 24 h postmortem. LT samples from the 12th to 13th ribs (2 cm × 3 cm × 5 cm, 25 ± 1 g) were weighed (initial weight) after removal of fat and moisture, then suspended in sealed inflated plastic bags (cold stored at 4 °C for 24 h), followed by reweighing (final weight). Drip loss percentage = (initial weight − final weight)/initial weight × 100% [13]. Within 45 min after slaughter, uniformly sized LT muscle samples of approximately 100 g were taken, placed in a steamer, and steamed with boiling water for 30 min. The muscle samples were then suspended on hooks to cool for 30 min, dried with filter paper, and reweighed. Cooking loss was determined by calculating weight change percentage. Within 45 min after slaughter, a piece of LT muscle sample measuring 6 cm × 3 cm × 3 cm was taken from the 7th to 9th ribs on the left side of the carcass and stored at 4 °C for 24 h. After being removed from the refrigerator, the meat sample was cooked to an internal temperature of 75 °C and then allowed to cool to room temperature. Subsequently, three cylindrical cores with a diameter of 1.27 cm were cut from each sample parallel to the fiber orientation of the muscle and sheared perpendicular to the fiber orientation. Shear force measurements were conducted using a texture analyzer (TA.XT. Plus, Godalming Stable Microsystems, Surrey, UK) equipped with a thin Warner–Bratzler blade with a V-shaped slot, with a blade thickness of 3.0 mm ± 0.2 mm, a test speed of 2.00 mm/s, and a trigger force of 0.0050 kg [14].

### 2.8. Serum Analysis

Concentrations of blood glucose (GLU), total protein (TP), albumin (ALB), blood urea nitrogen (BUN), total cholesterol (TC), and Triglyceride (TG) were determined by an automatic biochemical analyzer (Mindray BS-400 Chemical Analyzer, Mindray Biomedical Electronics Co., Ltd., Shenzhen, China). Concentrations of ceruloplasmin (Cp), glutathione peroxidase (GP), and Cu-Zn superoxide dismutase (CZ-SOD) were determined by assay kits (Shanghai Enzyme Union Biotechnology Co., Ltd., Shanghai, China).

### 2.9. Volatile Fatty Acids and Cellulase Activity

Concentrations of volatile fatty acids (VFAs) in the colonic digesta were measured as described by the previous report. Briefly, samples were thawed on ice, and approximately 0.5 g of the sample was added to 8 mL of deionized water. The mixture was then thoroughly homogenized by vortexing for 1 min and centrifuged at 13,000× *g* for 5 min. The 70 μL mixture was analyzed for VFA using a GC-System (Agilent Technologies 7890A-G3440A-GC System; Agilent Technologies, Santa Clara, CA, USA). In addition, cellulase activities (xylanase, carboxymethylcellulase, and β-glucanase) were measured by assay kits (Shanghai Enzyme Union Biotechnology Co., Ltd., Shanghai, China) [15].

### 2.10. DNA Extraction, 16S Sequence Processing, and Analysis

Microbial DNA was extracted from the fermentation samples using a E.Z.N.A.^®^ soil DNA Kit (Omega Biotechnology, Norcross, GA, USA) following the manufacturer’s instructions. DNA quality and concentration were assessed using gel electrophoresis and a NanoDrop^®^ ND-2000 spectrophotometer (Thermo Scientific Inc., Wilmington, NC, USA) and stored at −80 °C. The V3-V4 hypervariable regions of the bacterial 16S rRNA gene were amplified using primer pairs 338F and 806R on an ABI GeneAmp^®^ 9700 PCR thermocycler (ABI, Foster, CA, USA). Amplification consisted of 27 cycles of denaturation, annealing, extension, and the final step at 4 °C. As previously reported, the PCR products were purified, quantified, pooled, and sequenced on the Illumina MiSeq PE300 platform. Microbial data were analyzed using the Majorbio Cloud platform.

### 2.11. Statistical Analysis

Data were analyzed using the *Y_ijk_* = *µ* + *α_i_* + *β_j_* + *α_i_* × *β_j_* + *ε_ijk_* model, where *µ* was the population mean, *α_i_* was the effect of dietary fiber (*i* = 1, 2), *β_j_* was the effect of the dietary copper (*j* = 1, 2), *α_i_* × *β_j_* was the interaction between dietary fiber and dietary copper, and *ε_ijk_* was the residual effect. The data were analyzed using SPSS version 27.0 (IBM, Corp., Armonk, NY, USA). Dietary fiber, dietary copper, and interaction effects were analyzed with a two-way ANOVA. Differences between the 4 treatments were analyzed by multiple comparisons using the Tukey method. *p* < 0.05 was considered statistically significant. The results were expressed as the mean and standard error of the mean.

## 3. Results

### 3.1. Growth Performance

The final weight and ADG of finishing pigs in the low dietary fiber group were higher than those in the high fiber group (*p* < 0.05), while F:G was lower than that in the high dietary fiber group (*p* < 0.05; Table 3). When all four dietary conditions were compared, there were differences in the final weight, ADG, and F:G (*p* < 0.05), although no interaction effects were observed (*p* > 0.05). The final weight and ADG of finishing pigs in the HA group were lower than those in the LN and LS groups (*p* < 0.05). The F:G of finishing pigs in the LS group was lower than that in the HN and HS groups (*p* < 0.05).

### 3.2. Nutrients Utilization

The digestibility of DM, CP, CF, Ash, NDF, and DF of finishing pigs in the low dietary fiber group was higher than that in the high dietary fiber group (*p* < 0.05; Table 4). The digestibility of DM and CF of finishing pigs in the normal dietary copper group was lower than that in the supplemented dietary copper group (*p* < 0.05). When all four dietary conditions were compared, there were differences between one or more diets in the digestibility of DM, CP, CF, Ash, NDF, and DF (*p* < 0.05), but there were no interaction effects (*p* > 0.05). The digestibility of DM, CP, CF, Ash, NDF and DF in finishing pigs in the LS group was higher than that in the HN and HS groups (*p* < 0.05). The digestibility of CP in finishing pigs in the HN group was lower than that in the other three diet treatment groups (*p* < 0.05).

### 3.3. Blood Copper, Serum Biochemical Indicators, and Antioxidant Enzyme Activities

Blood TP concentration was higher in finishing pigs in the high dietary fiber group than in the low dietary fiber group (*p* < 0.05; Table 5). Blood Cu of finishing pigs in the normal dietary copper group was lower than that in the supplemented dietary copper group (*p* < 0.05). When all four dietary conditions were compared, there were differences between one or more diets in the blood Cu, GLU, and TP (*p* < 0.05), although no interaction effects were observed (*p* > 0.05). The blood Cu of finishing pigs in the LS group was higher than that in the LN and HN groups (*p* < 0.05). The GLU was higher in the LN group than in the HS group (*p* < 0.05). The TP of pigs in the HS group was higher than that in the LN and LS groups (*p* < 0.05).

### 3.4. Carcass Traits and Meat Quality

The BT and LA of finishing pigs in the low dietary fiber group were higher than those in the high dietary fiber group (*p* < 0.05; Table 6). When all four dietary treatments were compared, there were differences between one or more diets in the COL, LA, and a*_24h_ (*p* < 0.05), although no interaction effects were observed (*p* > 0.05). The COLs of finishing pigs in the HN and HS groups was lower than that in the LN group (*p* < 0.05). The HS and a*_24h_ of pigs in the HN group were lower than those in the LS group (*p* < 0.05).

### 3.5. Cellulase Activity in Colonic Digesta

The activities of carboxymethylcellulase and β-glucanase in colonic digesta of finishing pigs in high dietary fiber group were higher than those in low dietary fiber group (*p* < 0.05; Table 7). The carboxymethylcellulase activity of finishing pigs in the normal dietary copper group was lower than that in the supplemented dietary copper group (*p* < 0.05). When all four dietary treatments were compared, there were differences between one or more diets in the activities of carboxymethylcellulase and β-glucanase (*p* < 0.05), although no interaction effects were observed (*p* > 0.05). The activities of carboxymethylcellulase and β-glucanase in the colon of pigs in the HS group were higher than those in the other three diet treatment groups (*p* < 0.05).

### 3.6. Volatile Fatty Acids

The concentrations of acetic acid, propionic acid, and total volatile fatty acids (TVFAs) in colonic digesta of finishing pigs in the high dietary fiber group were higher than those in the low dietary fiber group (*p* < 0.05; Table 8). When all four dietary treatments were compared, there were differences between one or more diets in the concentrations of acetic acid, propionic acid, and TVFAs (*p* < 0.05), although no interaction effects were observed (*p* > 0.05). The concentration of acetic acid in the colonic digesta of finishing pigs in the HN and HS groups was higher than that in the LN group (*p* < 0.05). The concentration of propionic acid in the colonic digesta of the pigs in the LN group was lower than those in the LS, HN, and HS groups (*p* < 0.05). The concentration of TVFAs in the colonic chyme of the HN and HS groups was higher than that of the LN and LS groups (*p* < 0.05).

### 3.7. Gut Microbiota

**Figure 1:**
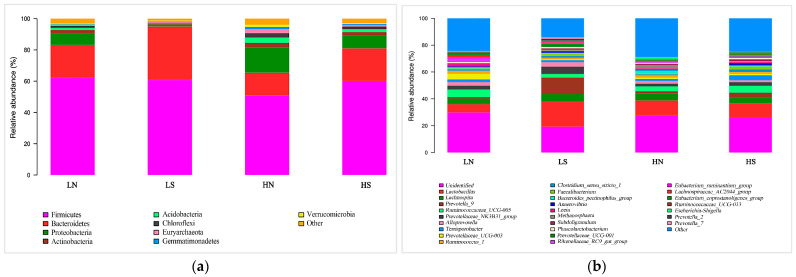
The colon microbial composition. (**a**) Relative abundance of major groups of cecal microorganisms at the phylum level. (**b**) Relative abundance of major groups of cecal microorganisms at the genus level.

The effects of dietary fiber and copper levels on microbial α-diversity indices in the colon of finishing pigs are shown in Table 9. The OB, Chao 1, and Shannon indices of colonic microorganisms of finishing pigs in the high dietary fiber group were higher than those in the low dietary fiber group (*p* < 0.05). Compared with the normal dietary copper level group, the dietary copper supplementation group decreased the OB, Chao 1, and Shannon indices of colon microorganisms (*p* < 0.05). The dietary fiber level and dietary copper level had an interaction effect on the α-diversity index of colonic microorganisms in finishing pigs (*p* < 0.05). The OB index of colonic microbes in the HN group was higher than that in the LN and LS groups (*p* < 0.05). The Chao 1 and Shannon indices of the colonic microorganisms in the LS group were lower than those in the LN, HN, and HS groups (*p* < 0.05). The microbial composition is shown in Figure 1. At the phylum level, the microbial composition in the colon of finishing pigs was mainly composed of *Firmicutes* (>58.5%) and *Bacteroidetes* (>22.7%). *Firmicutes*/*Bacteroidetes* ratios increased with the increase of the dietary fiber level. For *Bacteroidetes*, the relative abundance was higher in the LS group and lower in the HN group. Increasing the dietary fiber level increased the relative abundance of Proteobacteria in the colon of finishing pigs. However, increasing dietary copper levels decreased the relative abundance of Proteobacteria. At the genus level, the relative abundance of *Lactobacillus* was higher in the LS group and lower in the LN group. In addition, LS increased the relative abundance of *Prevotella* compared with other diet treatments.

## 4. Discussion

Dietary fiber and copper have different effects on the production performance of pigs. Previous studies have found that the growth-promoting effect of copper in pigs can be attributed to its role in enhancing intestinal development and improving the secretion of digestive enzymes in the small intestine, thereby increasing nutrient digestion and absorption [4]. However, this effect may be influenced by dietary fiber [3]. Therefore, we investigated the effects and interaction of dietary fiber and copper levels on the growth performance, nutrient utilization, carcass traits, meat quality, and gut microbiota of finishing pigs.

This study found that when the dietary fiber level was high (30%), the weight gain and feed conversion ratio (F:G) of finishing pigs were reduced. Guang et al. [16] reported that short-term supplementation of dietary fiber (16.7–17.75%) did not reduce the growth performance of finishing pigs, which is inconsistent with our findings. This discrepancy may be attributed to the higher fiber level (30%) and the longer feeding period (56 days) in our study. Notably, there was no difference in the average daily gain (ADG) of finishing pigs fed a high-fiber diet with copper supplementation compared to those fed a low-fiber diet, indicating that copper mitigated the negative impact of fiber on weight gain. This effect could be due to the positive role of copper in metabolism and growth, as it functions as a key cofactor for various metabolic enzymes and exhibits antibacterial properties [17].

In this study, the digestibility of DM, CP, CF, ash, NDF, and DF in finishing pigs decreased as the dietary fiber level increased, consistent with previous reports [16]. However, copper supplementation tended to alleviate the reduction in DM and CF digestibility induced by high fiber levels, following a pattern similar to those seen in earlier studies [18,19]. In this study, the digestibility of copper in finishing pigs was not negatively affected by dietary copper supplementation (45 mg/kg), indicating that appropriate copper supplementation did not lead to increased copper excretion. This outcome may be attributed to the ability of appropriate copper supplementation to improve the secretion of digestive enzymes in fattening pigs, which inhibits the binding interaction between carboxyl and hydroxyl groups in the fiber structure and divalent copper ions, thereby improving the deposition of trace elements in the body [20]. Therefore, appropriate copper supplementation in the diet of finishing pigs can mitigate the negative effects of increased fiber levels on nutrient digestibility.

The concentrations of blood urea and total protein are closely related to the absorption and utilization of protein in the body. Albumin functions in binding and transporting nutrients, including fatty acids, glucose, amino acids, and metal ions such as copper. This study demonstrated that a high dietary fiber level can increase the total protein content in pig blood, suggesting that an appropriate dietary fiber level may promote protein metabolism and absorption in the body [18]. Blood glucose is a key indicator reflecting carbohydrate and lipid metabolism. With the increase in dietary fiber level, a decreasing trend in blood glucose content in pigs was observed, consistent with our study results. This may be related to the viscosity of dietary fiber, which reduces the convection and diffusion rate of glucose in the digestive tract contents [18,21]. A reduction in blood glucose can lead to increased feed intake, aligning with growth performance indices. The conclusions regarding the effect of copper on blood glucose concentration are inconsistent. Our study found that blood glucose concentrations were not significantly affected across different groups, which may be due to variations in the source and level of copper and the growth stage of pigs [21]. Studies have shown that adequate copper supplementation in the diet can improve the antioxidant enzyme activity in pig blood [22]. Additionally, dietary fiber may improve the body’s antioxidant capacity by scavenging oxygen free radicals and inhibiting lipid peroxidation processes [23]. In this experiment, neither the dietary fiber level nor the copper level had an effect on the activity of antioxidant enzymes in the blood. This may be attributed to the limited range of dietary fiber and copper supplementation in this study.

Carcass weight, carcass length, and dressing percentage are important indicators for evaluating slaughter traits, while backfat thickness and loin-eye area reflect carcass lean percentage. Coble et al. [3] reported that replacing a corn–soybean meal-based diet with a high by-product diet (increasing NDF% from 7.35% to 14.95%) did not affect loin-eye area or backfat thickness, which is inconsistent with the results of our experiment. This discrepancy may be because the higher dietary fiber level diluted the energy and other nutrients in the diet, leading to an increase in feed intake as the body attempted to meet its nutritional needs. This resulted in an expansion of gastrointestinal volume and an increase in visceral organ mass, thus increasing the pre-slaughter live weight. Hong et al. [24] demonstrated that moderately reducing dietary fiber levels can increase the loin-eye area and backfat thickness in pigs, which is generally consistent with the findings of our study. Meat color is an important sensory indicator, with redness (a*) typically positively correlated with meat quality, while lightness (L*) and yellowness (b*) are negatively correlated with meat quality. Drip loss and shear force reflect the water-holding capacity and tenderness of meat, respectively, both of which are negatively correlated with meat quality [25]. Muscle pH is related to meat flavor, and pork pH generally declines to 5.6–6.0 within 24 h post-slaughter. Studies have shown that increasing the fiber level in finishing pig diets can significantly increase a* values and improve meat quality [26]. A higher dietary fiber level promotes the conversion of glycolytic muscle fibers to oxidative muscle fibers, with oxidative muscle fibers being positively correlated with a* values. This suggests that an appropriate fiber level can improve meat quality. However, no effect of fiber on meat quality was observed in this study, possibly due to the limited increase in fiber levels. Bai et al. [27] reported that supplementing appropriate levels of copper significantly increased the a* value of meat color and reduced drip loss, without adversely affecting pH or shear force, which is consistent with the results of our study. This outcome may be because copper participates in the synthesis of hemoglobin, with higher hemoglobin levels resulting in higher a* values. Oxidative muscle fibers rely on iron/copper-dependent proteins to maintain cellular activity. Research suggests that copper may improve meat color by increasing the number of oxidative muscle fibers and reducing the number of glycolytic muscle fibers, thereby increasing a* values [28].

The activity of dietary cellulase is closely related to the efficiency of fiber utilization in pigs. Studies have shown that high dietary fiber levels (40% alfalfa fiber) can induce an increase in the number and activity of fiber-degrading bacteria in the chyme of sows, thereby increasing cellulase and xylanase activity [29]. In this study, it was demonstrated that increasing the dietary fiber level increased the activity of colonic carboxymethyl cellulase and β-glucosidase in finishing pigs, which is consistent with the aforementioned findings. In addition, this is further consistent with the results of this experiment, where the supplementation of copper mitigated the decline in crude fiber and dry matter digestibility induced by high fiber. There are few studies on the effect of copper on cellulase activity in monogastric animals like pigs, but more research has been conducted on ruminants. For example, Wang et al. [30] reported that adding 10 mg/kg DM of copper to the diet of Holstein cows increased ruminal cellulase activity. Similarly, Zhao et al. [31] found that the cellulase activity in ruminal fluid increased with the rising copper concentration (5–25 mg/kg) in yak diets. In the present study, increasing the copper concentration from 25 mg/kg to 45 mg/kg elevated the carboxymethyl cellulase activity in the cecum of finishing pigs, which is consistent with the above research results. The reason may be that copper, as an essential trace element for fiber-degrading bacteria, not only promotes the proliferation of cellulose-degrading bacteria, thereby increasing the protein expression of fiber-degrading enzymes, but also enhances the biocatalytic reaction in the active center of cellulase, improving the efficiency of fiber degradation [32].

During microbial fermentation of fiber in the large intestine of pigs, the most abundantly produced short-chain fatty acids are acetate, propionate, and n-butyrate. Their production and concentration ratios depend on the type of microbial fermentation. Acetate impacts host energy and substrate metabolism by reducing fat breakdown, decreasing the number of pro-inflammatory cytokines, and increasing energy expenditure and fat oxidation, thereby influencing appetite [33]. Propionate serves as a substrate for hepatic gluconeogenesis, participating in the body’s metabolic processes [34,35], and it improves energy homeostasis by being converted into glucose through intestinal gluconeogenesis [36]. Studies have shown a positive effect between the VFA content in pig chyme and the dietary fiber level [37], which is consistent with the results of this study. Furthermore, research on ruminants has indicated that dietary copper supplementation can promote VFA production in the rumen of dairy cows [30]. In the present study, pigs fed a high-fiber diet supplemented with copper exhibited the highest VFA levels in their chyme, which corresponded with the fiber digestibility results. The likely reason is that a high-fiber level provides ample fermentable substrates for microorganisms, while elevated copper levels may induce microbial proliferation and enhance the efficiency of synthesizing fiber-degrading enzymes.

The gut microbiota plays a critical role in influencing nutrient digestion and absorption, maintaining physiological homeostasis, promoting immune system development, regulating host metabolism, and inhibiting exogenous pathogenic microorganisms [38,39,40]. In this study, increasing dietary fiber levels elevated the OTU count, Chao 1 index, and Shannon index in the colon of pigs, consistent with previous research findings [41]. Studies have shown that copper supplementation in the diet can regulate the microbiota, thereby reducing potential intestinal pathogens [42]. Research also suggests that high copper levels can decrease the Chao 1 and Shannon indices in the large intestine of finishing pigs, thus affecting microbial richness [43]. The impact of copper on microbial cells is related to the induction of intracellular ROS, which inactivate relevant cellular components and lead to microbial death [44]. However, bacteria can develop copper tolerance by reducing permeability, utilizing metallothioneins (copper export proteins) in the cytoplasm and periplasm, and employing active copper transport mechanisms [43]. Therefore, dietary copper levels can induce changes in microbial population and structure.

This study found that under low-fiber diets (compared to high-fiber diets), the reduction in microbial α-diversity due to copper supplementation was further amplified. This suggests that adjustments to copper dosage should be considered when dietary fiber levels differ. Additionally, this study revealed that *Firmicutes* and *Bacteroidetes* were the dominant phyla in high-fiber diets, and the relative abundance ratio of *Firmicutes* to *Bacteroidetes* (F/B) increased with higher fiber levels, indicating that high-fiber diets promoted the enrichment of fiber-degrading bacteria [45]. Studies have shown that dietary fiber levels can increase the relative abundance of Lactobacillus, inhibit harmful bacteria, and optimize the microbial community [46]. In this study, both dietary fiber and copper levels increased the relative abundance of *Lactobacillus*, which aligns with the findings of previous research. In this study, increasing the copper concentration increased the relative abundance of *Prevotella*. *Prevotella* is known to produce fiber-degrading enzymes to break down dietary fibers such as cellulose and cellobiose [47], and ferment them to produce acetic acid, propionic acid, and succinic acid, which reduce intestinal pH and are crucial for the development of the intestinal barrier and immune system [48]. The current experiment found that increasing both dietary fiber levels and copper concentration increased the abundance of most fiber-degrading bacteria, which is beneficial for dietary fiber degradation. Dietary fiber can serve as a substrate for microbial fermentation, promoting the proliferation of fiber-degrading bacteria, and generating volatile fatty acids through fiber fermentation. In high-fiber dietary conditions, finishing pigs supplemented with copper showed higher levels of various short-chain fatty acids in their colon compared to the control group, further aligning with the results of the pigs’ growth performance. Compared to high-fiber treatments, copper supplementation in low-fiber diets further improved the relative abundance of *Lactobacillus* and *Prevotella*, suggesting that the regulatory effects of copper on the microbial community should be considered when dietary fiber levels are elevated.

In summary, this study found that high-fiber diets reduced the growth performance, nutrient digestibility, and carcass traits of finishing pigs, while modulating the microbial community, increasing fiber-degrading enzyme activity, and elevating microbial metabolites. Copper supplementation in the diets of finishing pigs showed a trend of improving nutrient digestibility, affecting the gut microbiota, and increasing cellulase activity. Furthermore, copper supplementation in high-fiber diets mitigated the negative effects on the production performance of finishing pigs and had an interactive effect on the regulation of the microbial community.

## 5. Conclusions

Supplementing appropriate levels of copper in high-fiber diets for finishing pigs does not lead to excessive copper excretion. Additionally, adequate copper can alleviate the reduction in production performance induced by high fiber levels by increasing nutrient digestibility, enhancing fiber-degrading enzyme activity, and regulating microbial flora and their metabolites.

## Figures and Tables

**Table 1:** Ingredient composition analysed nutrient contents of feedstuff.

Item	Analyzed Nutrient Composition (n = 6, Dry Matter Basis, %)								
	Corn	Soybean Meal	Alfalfa Meal	Sugar Beet Pulp	DDGS	Corn Gluten Meal	Corn Bran	Soybean Hulls	Canola Meal
Metabolizable energy, MJ/kg ^A^	14.35 ± 0.54	15.26 ± 0.44	5.87 ± 0.20	12.26 ± 0.39	12.98 ± 0.27	18.92 ± 0.59	9.06 ± 0.32	10.69 ± 0.78	10.28 ± 0.49
DM, % ^B^	85.60 ± 5.33	94.88 ± 4.98	90.03 ± 4.25	88.14 ± 5.43	90.91 ± 1.06	97.34 ± 3.92	95.25 ± 3.8	90.11 ± 2.69	93.39 ± 2.76
Crude Protein, % ^B^	8.45 ± 0.23	44.96 ± 2.32	16.32 ± 0.60	9.09 ± 0.45	26.88 ± 1.19	62.69 ± 3.02	15.44 ± 0.35	10.49 ± 0.61	36.04 ± 1.88
Ether extract, % ^B^	2.12 ± 0.20	5.89 ± 0.07	21.83 ± 0.02	17.65 ± 0.04	6.79 ± 0.22	2.52 ± 0.02	14.71 ± 0.18	36.36 ± 0.07	13.05 ± 0.12
Ash, % ^B^	0.13 ± 0.00	0.15 ± 0.00	0.31 ± 0.01	0.70 ± 0.04	0.14 ± 0.00	0.16 ± 0.00	0.12 ± 0.01	0.23 ± 0.01	0.36 ± 0.02
Crude fiber, % ^B^	1.99 ± 0.11	5.74 ± 0.24	21.52 ± 0.80	16.95 ± 0.43	6.64 ± 0.27	2.36 ± 0.07	14.59 ± 0.43	36.14 ± 1.27	12.69 ± 0.6
Calcium, % ^B^	0.02 ± 0.00	0.40 ± 0.02	1.18 ± 0.01	0.80 ± 0.06	0.1 ± 0.00	0.02 ± 0.00	0.15 ± 0.01	1.00 ± 0.07	0.75 ± 0.04
Total Phosphorus, % ^B^	0.57 ± 0.02	0.64 ± 0.04	0.30 ± 0.01	0.15 ± 0.01	0.72 ± 0.03	0.09 ± 0.00	0.50 ± 0.03	0.29 ± 0.01	0.90 ± 0.04
Available Phosphorus, % ^A^	0.15 ± 0.00	0.27 ± 0.00	0.15 ± 0.01	0.16 ± 0.01	0.47 ± 0.01	0.03 ± 0.00	0.17 ± 0.01	0.08 ± 0.01	0.41 ± 0.02
Lysine, % ^B^	0.25 ± 0.02	2.76 ± 0.10	0.73 ± 0.03	0.51 ± 0.02	0.76 ± 0.03	0.82 ± 0.02	0.50 ± 0.01	0.66 ± 0.03	1.95 ± 0.10
Methionine, % ^B^	0.18 ± 0.01	0.62 ± 0.03	0.25 ± 0.01	0.07 ± 0.00	0.47 ± 0.01	1.34 ± 0.02	0.25 ± 0.01	0.11 ± 0.01	0.75 ± 0.05
Cysteine, % ^B^	0.21 ± 0.01	0.64 ± 0.03	0.18 ± 0.01	0.06 ± 0.00	0.41 ± 0.02	1.08 ± 0.03	0.40 ± 0.01	0.20 ± 0.01	0.98 ± 0.05
Threonine, % ^B^	0.28 ± 0.02	1.70 ± 0.08	0.71 ± 0.02	0.38 ± 0.01	1.00 ± 0.04	1.70 ± 0.04	0.61 ± 0.04	0.37 ± 0.01	1.56 ± 0.09
Tryptophan, % ^B^	0.06 ± 0.00	0.58 ± 0.02	0.24 ± 0.01	0.10 ± 0.01	0.21 ± 0.01	0.23 ± 0.01	0.07 ± 0.00	0.09 ± 0.01	0.46 ± 0.03

Values are least-squares mean values ± standard error. ^A^ Calculated according to NRC (2012). ^B^ The contents of DM, Crude Protein, Ether extract, Ash, Crude fiber, Calcium, Total Phosphorus, Lysine, Methionine, Cysteine, Threonine, and Tryptophan were analyzed.

**Table 2:** Composition and nutrient levels of the diet (60–100 kg).

Ingredients, %	Factors and Treatments ^A^			
	LN	LS	HN	HS
Corn	49.49	49.48	36.60	36.59
Soybean meal	8.00	8.00	9.50	9.50
Alfalfa meal	1.50	1.50	10.00	10.00
Sugar beet pulp	16.50	16.50	18.50	18.50
DDGS	2.00	2.00	5.00	5.00
Corn gluten meal	0.50	0.50	2.00	2.00
Corn bran	0.00	0.00	4.00	4.00
Soybean hulls	0.00	0.00	4.50	4.50
Canola meal	14.50	14.50	2.00	2.00
Soya-bean oil	4.00	4.00	5.00	5.00
CuSO_4_·5H_2_O	0.010	0.018	0.010	0.018
NaCl	0.35	0.35	0.35	0.35
Stone dust	1.15	1.15	1.10	1.10
CaPH_4_	0.50	0.50	0.30	0.30
L-Lys monohydrochloride	0.35	0.35	0.05	0.05
L-Met monohydrochloride	0.00	0.00	0.05	0.05
L-Thr monohydrochloride	0.08	0.08	0.00	0.00
L-Trp monohydrochloride	0.07	0.07	0.04	0.04
Premix ^B^	1.00	1.00	1.00	1.00
Total	100.00	100.00	100.00	100.00
Nutrient levels				
Metabolizable energy (MJ/kg) ^C^	13.47	13.47	13.47	13.47
Crude protein, SID % ^C^	10.51	10.49	10.51	10.51
Dietary fiber % ^D^	23.6	23.98	30.7	30.7
Calcium% ^D^	0.75	0.78	0.78	0.78
Total phosphorus % ^D^	0.57	0.54	0.53	0.52
Available phosphorus % ^C^	0.24	0.24	0.23	0.23
Lysine % ^D^	0.71	0.69	0.71	0.71
Methionine + Cysteine % ^D^	0.56	0.58	0.55	0.55
Threonine % ^D^	0.55	0.65	0.56	0.56
Tryptophan % ^D^	0.19	0.21	0.19	0.19
Cu, mg/kg ^D^	26.84	46.56	27.22	47.74

^A^ LN: diet with DF level (23%) and Cu (25 mg/kg); LS: diet with DF level (23%) and Cu (45 mg/kg); HN: diet with DF level (30%) and Cu (25 mg/kg); and HS: diet with DF level (30%) and Cu (45 mg/kg). ^B^ The premix provided the following per kilogram of diets: 0.20 mg KI; 400 mg FeSO_4_·7H_2_O; 0.56 mg NaSeO_3_; 359 mg ZnSO_4_·7H_2_O; 10.2 mg MnSO_4_·H_2_O; 5 mg vitamin K (menadione); 2 mg vitamin B_1_; 15 mg vitamin B_2_; 30 μg vitamin B_12_; 5400 IU vitamin A; 110 IU vitamin D_3_; 18 IU vitamin E; 80 mg choline chloride; 20 mg antioxidants; and 100 mg Fungicide. ^C^ Calculated according to NRC (2012). ^D^ Measured value.

**Table 3:** Effects of dietary fiber and Cu levels on growth performance in finishing pigs.

Items	DF Level (%)		Cu (mg/kg)		Treatment				SEM	*p* Value		
	L (23%)	H (30%)	N (25 mg/kg)	S (45 mg/kg)	LN	LS	HN	HS		DF	Cu	CF × Cu
Initial weight (kg)	76.55	76.35	76.51	76.41	76.25	76.85	76.75	75.95	0.295	0.752	0.868	0.273
Final weight (kg)	123.55 ^a^	119.38 ^b^	120.36	122.57	122.70 ^a^	124.42 ^a^	118.04 ^b^	120.73 ^ab^	0.668	0.012	0.129	0.721
ADFI (kg/d)	2.95	2.79	2.83	2.78	2.98	2.93	2.69	2.88	0.062	0.168	0.538	0.322
ADG (kg/d)	0.84 ^a^	0.77 ^b^	0.79	0.83	0.83 ^a^	0.85 ^a^	0.74 ^b^	0.80 ^ab^	0.009	0.018	0.158	0.523
F:G	3.50 ^b^	3.63 ^a^	3.62	3.53	3.58 ^ab^	3.45 ^b^	3.65 ^a^	3.61 ^a^	0.029	0.042	0.105	0.383

Means within the same row for the individual effects of DF level, Cu, and treatments with different superscript letters differ (*p* < 0.05). ADFI: average daily feed intake; ADG: average daily gain; F:G: the ratio of feed to gain.

**Table 4:** Effects of dietary fiber and Cu levels on nutrient digestibility in finishing pigs.

Items	DF Level (%)		Cu Level (mg/kg)		Treatment				SEM	*p* Value		
	L (23%)	H (30%)	N (25 mg/kg)	S (45 mg/kg)	LN	LS	HN	HS		DF	Cu	CF × Cu
DM (%)	84.66 ^a^	82.67 ^b^	82.94 ^b^	84.25 ^a^	83.99 ^ab^	85.21 ^a^	82.07 ^c^	83.28 ^bc^	0.303	<0.001	0.052	0.981
EE (%)	79.61	80.67	79.44	80.97	78.66	80.87	80.21	81.03	0.453	0.362	0.123	0.462
CP (%)	83.11 ^a^	80.21 ^b^	81.27	82.01	82.56 ^ab^	83.57 ^a^	79.97 ^c^	80.44 ^b^	0.412	<0.001	0.750	0.387
CF (%)	44.59 ^a^	41.56 ^b^	42.18 ^b^	43.77 ^a^	43.57 ^ab^	45.45 ^a^	41.03 ^c^	42.09 ^bc^	0.393	<0.001	0.071	0.604
Ash (%)	40.86 ^a^	37.87 ^b^	38.85	39.88	40.19 ^ab^	41.53 ^a^	37.51 ^c^	38.23 ^bc^	0.379	0.001	0.194	0.690
NDF (%)	45.65 ^a^	41.99 ^b^	42.52	44.65	44.04 ^ab^	47.26 ^a^	41.50 ^b^	42.57 ^b^	0.716	0.021	0.152	0.462
ADF (%)	41.36	39.44	39.79	40.97	40.52	42.21	39.21	39.73	0.567	0.120	0.341	0.613
DF (%)	45.65 ^a^	41.99 ^b^	42.52	44.65	44.04 ^ab^	47.26 ^a^	41.50 ^b^	42.57 ^b^	0.714	0.023	0.152	0.460
Cu (%)	52.15	49.87	51.55	50.48	52.78	50.32	48.92	49.43	0.548	0.054	0.340	0.864

Means within the same row for the individual effects of DF level, Cu, and treatments with different superscript letters differ (*p* < 0.05). DM: dry matter, EE: ether extract, CP: crude protein, NDF: neutral detergent fiber, ADF: acid detergent fiber, and DF: dietary fiber.

**Table 5:** Effects of dietary fiber and Cu levels on Blood biochemical and antioxidant enzyme activity in finishing pigs.

Items	DF Level (%)		Cu Level (mg/kg)		Treatment				SEM	*p* Value		
	L (23%)	H (30%)	N (25 mg/kg)	S (45 mg/kg)	LN	LS	HN	HS		DF	Cu	DF × Cu
Blood Cu (mg/L)	1.72	1.64	1.62 ^b^	1.74 ^a^	1.65 ^b^	1.81 ^a^	1.59 ^b^	1.69 ^ab^	0.017	0.078	0.009	0.582
Blood biochemical parameters												
GLU (mmol/L)	4.27	3.77	4.13	3.9	4.29 ^a^	4.24 ^ab^	3.98 ^ab^	3.55 ^b^	0.113	0.066	0.330	0.423
TP (g/L)	69.10 ^b^	72.77 ^a^	69.87	72	68.70 ^b^	69.50 ^b^	71.03 ^ab^	74.50 ^a^	0.764	0.043	0.200	0.412
ALB (g/L)	39.27	41.48	39.5	41.25	38.6	39.93	40.4	42.57	1.156	0.364	0.470	0.863
UREA (mmol/L)	4.97	4.67	4.87	4.77	5.01	4.93	4.73	4.61	0.074	0.093	0.550	0.894
TC (mmol/L)	3.27	3.11	3.15	3.2	3.26	3.28	3.03	3.11	0.093	0.284	0.780	0.923
TG (mmol/L)	1.12	0.92	0.96	1.08	1.04	1.2	0.88	0.96	0.143	0.495	0.680	0.892
Blood antioxidant enzyme activity												
Cp (ug/mL)	297.76	290.35	286.77	301.34	291.92	303.6	281.62	299.08	7.213	0.616	0.320	0.843
GSH-PX (pmol/mL)	71.61	68.19	68.42	71.37	69.98	73.23	66.86	69.51	1.144	0.154	0.220	0.904
CuZn-SOD (pg/mL)	32.9	31.58	31.75	32.73	32.43	33.38	31.08	32.09	0.545	0.253	0.384	0.973

Means within the same row for the individual effects of DF level, Cu, and treatments with different superscript letters differ (*p* < 0.05). GLU: glucose, TP: total protein, ALB: albumin, TC: total cholesterol, TG: triglycerides, Cp: ceruloplasmin, GSH-PX: glutathione peroxidase, and CuZn-SOD: copper–zinc superoxide dismutase.

**Table 6:** Effects of dietary fiber and Cu levels on carcass traits and meat quality in finishing pigs.

Items	DF Level (%)		Cu Level (mg/kg)		Treatment				SEM	*p* Value		
	L (23%)	H (30%)	N (25 mg/kg)	S (45 mg/kg)	LN	LS	HN	HS		DF	Cu	DF × Cu
Carcass traits												
HCW (kg)	82.07	80.80	80.87	82.00	81.26	82.74	80.35	81.4	0.590	0.312	0.363	0.862
DP (%)	71.80	70.25	70.55	71.50	71	72.01	69.51	71.6	0.681	0.274	0.503	0.705
COL (cm)	100.56	99.08	99.50	100.14	100.13 ^ab^	101.00 ^a^	98.88 ^b^	99.28 ^ab^	0.350	0.058	0.382	0.740
BT (mm)	2.22 ^a^	2.36 ^b^	2.31	2.27	2.24	2.2	2.38	2.33	0.028	0.048	0.509	0.862
LA (cm^2^)	49.93 ^a^	48.72 ^b^	49.11	49.54	49.79 ^ab^	50.07 ^a^	48.43 ^b^	49.02 ^ab^	0.251	0.033	0.387	0.763
Meat quality												
Lightness (L*)_24h_	49.77	47.95	49.14	48.59	49.84	49.7	48.43	47.47	0.531	0.114	0.622	0.712
Redness (a*)_24h_	7.60	8.20	7.73	8.16	7.60 ^b^	7.76 ^ab^	7.85 ^ab^	8.55 ^a^	0.007	0.092	0.153	0.364
Yelowness (b*)_24h_	9.27	8.96	9.19	9.03	9.34	9.19	9.05	8.87	0.078	0.123	0.403	0.918
Cooking loss (%)	33.15	32.65	32.80	33.00	33.10	33.20	32.50	32.80	0.371	0.283	0.632	0.703
Drip Loss (%)	2.19	2.10	2.15	2.13	2.21	2.16	2.10	2.10	0.018	0.078	0.623	0.542
pH_24h_	5.74	5.81	5.75	5.79	5.74	5.73	5.76	5.86	0.028	0.163	0.412	0.283
Shear force (N)	45.28	43.63	44.12	44.79	44.79	45.77	43.46	43.81	0.512	0.204	0.593	0.805

Means within the same row for the individual effects of DF level, Cu, and treatments with different superscript letters differ (*p* < 0.05). HCW: hot carcass weight, DP: dressing percentage, COL: carcass oblique length, BT: backfat thickness, and LA: loin-eye area.

**Table 7:** Effects of dietary fiber and Cu levels on colonic digesta cellulase activity in finishing pigs.

Items	DF Level (%)		Cu Level (mg/kg)		Treatment				SEM	*p* Value		
	L (23%)	H (30%)	N (25 mg/kg)	S (45 mg/kg)	LN	LS	HN	HS		DF	Cu	DF × Cu
Xylanase (pg/mL)	437.05	444.78	433.96	447.87	431.73	442.36	436.19	453.38	5.208	0.486	0.222	0.763
CMC (pg/mL)	320.87 ^b^	349.96 ^a^	325.92 ^b^	344.91 ^a^	315.21 ^b^	326.53 ^b^	336.63 ^b^	363.29 ^a^	3.360	<0.001	0.021	0.293
β-glu (ng/L)	82.56 ^b^	88.03 ^a^	83.75	86.84	82.42 ^b^	82.69 ^b^	85.07 ^b^	90.98 ^a^	0.900	0.023	0.119	0.162

Means within the same row for the individual effects of DF level, Cu, and treatments with different superscript letters differ (*p* < 0.05). CMC: Carboxymethyl Cellulose and β-glu: β-glucosidase.

**Table 8:** Effects of dietary fiber and Cu levels on colonic digesta volatile fatty acids in finishing pigs.

Items	DF Level (%)		Cu Level (mg/kg)		Treatment				SEM	*p* Value		
	L (23%)	H (30%)	N (25 mg/kg)	S (45 mg/kg)	LN	LS	HN	HS		DF	Cu	DF × Cu
Acetic acid (mmol/L)	63.55 ^b^	73.62 ^a^	68.33	70.56	61.47 ^b^	65.64 ^ab^	72.90 ^a^	74.49 ^a^	1.449	<0.001	0.343	0.658
Propionic acid (mmol/L)	19.69 ^b^	25.56 ^a^	21.17	24.11	17.54 ^b^	21.83 ^a^	25.26 ^a^	25.93 ^a^	0.712	<0.01	0.081	0.218
Butyric acid (mmol/L)	9.32	10.41	9.5	10.46	8.57	10.08	10.11	10.76	0.479	0.272	0.282	0.657
TVFAs (mmol/L)	92.56 ^b^	109.60 ^a^	100	105.12	87.59 ^b^	97.54 ^b^	108.27 ^a^	111.18 ^a^	1.781	<0.01	0.078	0.332

Means within the same row for the individual effects of DF level, Cu, and treatments with different superscript letters differ (*p* < 0.05). TVFAs = Total volatile fatty acids.

**Table 9:** The effects of dietary fiber and copper levels on microbial α-diversity indices in the colon of finishing pigs.

Items	DF Level (%)		Cu Level (mg/kg)		Treatment				SEM	*p* Value		
	L (23%)	H (30%)	N (25 mg/kg)	S (45 mg/kg)	LN	LS	HN	HS		DF	Cu	DF × Cu
observed_species	1986.87 ^b^	3134.73 ^a^	2974.65 ^a^	2146.95 ^b^	2629.80 ^b^	1343.93 ^c^	3319.50 ^a^	2949.97 ^ab^	98.890	<0.01	<0.01	0.05
chao1	2957.10 ^b^	4221.87 ^a^	4063.72 ^a^	3115.25 ^b^	3798.37 ^a^	2115.84 ^b^	4329.07 ^a^	4114.66 ^a^	148.710	<0.01	0.01	0.04
shannon	7.41 ^b^	8.47 ^a^	8.54 ^a^	7.34 ^b^	8.36 ^a^	6.46 ^b^	8.72 ^a^	8.22 ^a^	0.150	0.01	<0.01	0.05

Means within the same row for the individual effects of DF level, Cu, and treatments with different superscript letters differ (*p* < 0.05).

## Data Availability

The original contributions presented in this study are included in this article, further inquiries can be directed to the corresponding authors.

## Abbreviations

ADFI: average daily feed intake; ADG: average daily gain; F:G: the ratio of feed to gain; DM: dry matter; EE: ether extract; CP: crude protein; NDF: neutral detergent fiber; ADF: acid detergent Fiber; DF: dietary fiber; GLU: glucose; TP: total protein; ALB: albumin, TC: total cholesterol; TG: triglycerides; Cp: ceruloplasmin; GSH-PX: glutathione peroxidase; CuZn-SOD: copper–zinc superoxide dismutase; HCW: hot carcass weight; DP: dressing percentage; COL: carcass oblique length; BT: backfat thickness; LA: loin-eye area; CMC: carboxymethyl cellulose; β-glu: β-glucosidase; TVFAs: total volatile fatty acids.
